# Membrane-Disruptive Effects of Fatty Acid and Monoglyceride Mitigants on *E. coli* Bacteria-Derived Tethered Lipid Bilayers

**DOI:** 10.3390/molecules29010237

**Published:** 2024-01-01

**Authors:** Sue Woon Tan, Bo Kyeong Yoon, Joshua A. Jackman

**Affiliations:** 1 School of Chemical Engineering and Translational Nanobioscience Research Center, Sungkyunkwan University, Suwon 16419, Republic of Korea; 2 School of Healthcare and Biomedical Engineering, Chonnam National University, Yeosu 59626, Republic of Korea

**Keywords:** antimicrobial, fatty acid, monoglyceride, tethered bilayer lipid membrane, critical micelle concentration, electrochemical impedance spectroscopy

## Abstract

We report electrochemical impedance spectroscopy measurements to characterize the membrane-disruptive properties of medium-chain fatty acid and monoglyceride mitigants interacting with tethered bilayer lipid membrane (tBLM) platforms composed of *E. coli* bacterial lipid extracts. The tested mitigants included capric acid (CA) and monocaprin (MC) with 10-carbon long hydrocarbon chains, and lauric acid (LA) and glycerol monolaurate (GML) with 12-carbon long hydrocarbon chains. All four mitigants disrupted *E. coli* tBLM platforms above their respective critical micelle concentration (CMC) values; however, there were marked differences in the extent of membrane disruption. In general, CA and MC caused larger changes in ionic permeability and structural damage, whereas the membrane-disruptive effects of LA and GML were appreciably smaller. Importantly, the distinct magnitudes of permeability changes agreed well with the known antibacterial activity levels of the different mitigants against *E. coli*, whereby CA and MC are inhibitory and LA and GML are non-inhibitory. Mechanistic insights obtained from the EIS data help to rationalize why CA and MC are more effective than LA and GML at disrupting *E. coli* membranes, and these measurement capabilities support the potential of utilizing bacterial lipid-derived tethered lipid bilayers for predictive assessment of antibacterial drug candidates and mitigants.

## 1. Introduction

The design of cell-membrane-mimicking lipid bilayer platforms is of high interest for understanding the structure and function of cell membranes, as well as for applications such as pharmaceutical drug discovery and biosensor development [1,2]. Recently, reconstructing bacterial lipid membranes on sensing devices has become an important area for investigating bacteria–material interactions as well as for scrutinizing biomacromolecular interaction processes like enzymatic and antibiotic activities that occur at bacterial cell membrane interfaces [3,4,5]. As such, the development of well-defined bacterial lipid membrane model systems for different bacterial species is essential because each one has a particular lipid composition, which can even vary among strains [6]. By utilizing purified lipid extracts derived from bacterial cell membranes, it is possible to incorporate the wide range of naturally occurring lipids, which is advantageous for attempting to mimic membrane properties such as fluidity and rigidity [7,8,9,10]. Thus far, the lipid extract of *Escherichia coli* (*E. coli*) bacteria has been the most commonly used model system due to its commercial availability and the importance of *E. coli* as an important bacterial species relevant to human and animal health and to food safety [11]. In combination with biosensing techniques, it is thus possible to study how different types of antimicrobial drug candidates and mitigants interact with *E. coli* membranes and to define potencies and mechanisms of action [3,12]. 

The most popular technique for obtaining lipid extracts from *E. coli* bacterial cells is solvent extraction, and the total lipid mass can be extracted using a modified Bligh and Dyer method [13,14,15]. These *E. coli* lipid extracts contain zwitterionic phosphatidylethanolamine (PE), negatively charged phosphatidylglycerol (PG), and doubly negatively charged cardiolipin (CL) lipids as the major components, and can be used to fabricate bacterial lipid membranes on sensor surfaces. The inclusion of CL, in particular, is an important advantage of working with *E. coli* lipid extracts compared to more simplified, binary PE/PG lipid model compositions because the four-chain CL lipid has been reported to have a strong influence on membrane organization [16,17]. In addition to studying membrane permeabilization of *E. coli* lipid vesicles in bulk solution [18], different types of solid-supported lipid membrane platforms composed of purified *E. coli* lipids have been developed depending on the sensing application and mainly involve the surface deposition of lipid vesicles prepared from *E. coli* lipids. For instance, the adsorption and spontaneous rupture of *E. coli* lipid vesicles to form supported lipid bilayers (SLBs) on gold, titania, and silica surfaces has been reported by modulating the vesicle–surface interaction strength in different solution environments [9,19,20,21,22]. It has also been possible to form an intact adlayer of unruptured *E. coli* lipid vesicles on solid surfaces in order to mimic the curved surface of *E. coli* bacterial cell membranes [23].

Another promising platform is the tethered bilayer lipid membrane (tBLM) that can be fabricated on gold electrode surfaces by using *E. coli* lipid extracts based on a rapid-solvent-exchange-type process and is compatible with electrochemical impedance spectroscopy (EIS) to characterize the electrochemical properties of *E. coli* tBLMs, which are related to membrane integrity. For example, Berry et al. employed *E. coli* tBLM platforms in conjunction with EIS measurements to study the interaction kinetics of how a cationic antimicrobial peptide and engineered versions thereof disrupt the bacterial cell membrane mimic [24]. Notably, a degree of correlation was shown between the ability of a peptide to increase ionic permeability across the *E. coli* tBLM and in vitro antibacterial activity level [24]. It is important to further expand such capabilities to evaluate the membrane-disruptive properties of antimicrobial lipids, especially medium-chain fatty acids and monoglycerides, that are important mitigants for food safety and agricultural applications [25,26]. Interestingly, while various fatty acid and monoglyceride mitigants within this class exhibit potent antibacterial properties based on permeabilization-related membrane disruption [27], only a subset of them inhibits *E. coli* bacteria and elucidating the biophysical basis for these different targeting spectrums is an outstanding need that can benefit from *E. coli* tBLM platforms.

**Figure 1:**
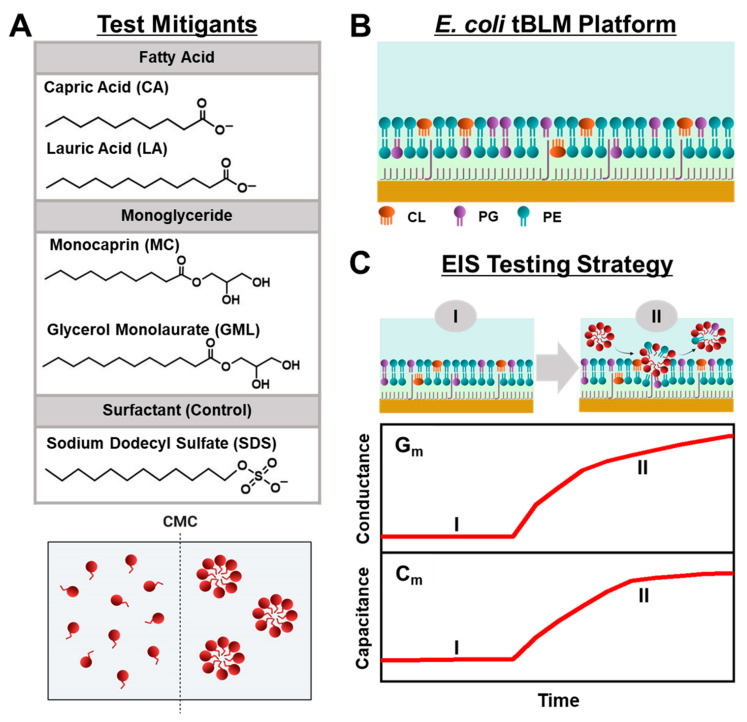
Overview of EIS-based antibacterial-mitigant-testing strategy. (**A**) Molecular structures of medium-chain fatty acids and monoglycerides (CA, LA, MC, GML) and surfactant control (SDS). These amphipathic molecules are dispersed as free monomers below their respective CMC values but begin self-assembling to form micelles at and above their respective CMC values. (**B**) Schematic illustration of tBLM platform consisting of reconstituted *E. coli* lipid extract that contains CL, PG, and PE lipids among various components. (**C**) Measurement concept based on (I) *E. coli* tBLM fabrication and (II) subsequent addition of test compound at defined bulk concentration. Time-resolved EIS measurements were performed to track changes in the electrical conductance (G_m_) and capacitance (C_m_) properties of the *E. coli* tBLM platform during the interaction process. The presented graphs are schematic illustrations for conceptual purposes and, thus, unitless without dimensions.

Herein, we conducted EIS measurements to comparatively evaluate the membrane-disruptive effects of medium-chain fatty acid and monoglyceride mitigants on *E. coli* tBLM platforms. Our approach builds on recent efforts to study antimicrobial lipid and detergent interactions with simplified tBLM platforms [28,29,30] and extends the measurement concept to investigate how various biologically important antimicrobial mitigants interact with reconstituted *E. coli* lipid membranes. As depicted in Figure 1A, we selected two pairs of medium-chain fatty acids and monoglycerides—the 10-carbon long capric acid (CA) fatty acid and its monoglyceride equivalent monocaprin (MC), and the 12-carbon long lauric acid (LA) fatty acid and its monoglyceride equivalent glycerol monolaurate (GML)—for EIS testing because these mitigants are among the most potent, membrane-disruptive ones against Gram-positive bacteria [31], yet have varying levels of antibacterial activity against Gram-negative bacteria such as *E. coli*. From a chemical perspective, all four mitigants are amphipathic molecules that self-assemble into micelles in bulk solution above their respective critical micelle concentration (CMC) values, whereas they exist as monomers at lower concentrations. Using the EIS technique, we conducted concentration-dependent experiments to investigate how different concentrations of each mitigant induce real-time changes in the conductance (G_m_) and capacitance (C_m_) properties of *E. coli* tBLM platforms that are sensitive to membrane ionic permeability and structural integrity, respectively [32] (Figure 1B,C). Our findings demonstrate that EIS measurements on *E. coli* tBLM platforms are a versatile tool to directly test the membrane-disruptive properties of fatty acid and monoglyceride mitigants and show how ionic permeability changes can be related to the antibacterial activity of different mitigants in terms of both potency and disruption effect magnitude. 

## 2. Results and Discussion

We focused on fabricating tBLM platforms composed of total *E. coli* lipid extract, and subsequently investigated the membrane-disruptive effects of 10- and 12-carbon long fatty acids and monoglycerides on these bacterial cell membrane mimics. This approach was based on measuring changes in membrane conductance and capacitance with the EIS technique, and we tested each compound at bulk concentrations corresponding to 4× and 2× of their respective CMC values. In general, shorter-chain fatty acids and monoglycerides have higher CMC values because they are more soluble and have less thermodynamic propensity to self-assemble into micelles [33]. 

The tBLM platforms were fabricated on functionalized gold electrode surfaces by using the rapid-solvent-exchange method and possess an ionic reservoir (~4 nm thickness) between the tBLM bottom leaflet and gold electrode surface, as previously described [34,35]. Operationally, the frequency-dependent impedance and phase properties of the fabricated tBLM platforms in aqueous buffer solution were measured by the EIS technique (see Figure S1 for representative Bode and Nyquist plots [36] of an *E. coli* tBLM platform formed using total lipid extract). A frequency sweep was performed to collect impedance and phase data across the full frequency range once every ~3 min cycle and fitted to an equivalent circuit model to monitor time-resolved changes in G_m_ and C_m_ signals, which are related to tBLM conductance (i.e., ionic permeability) and capacitance (i.e., structural integrity), respectively. The corresponding values were determined to be in the range of ~1–2 μS and 0.5–1.1 μF/cm^2^, respectively, which indicate that the fabricated tethered lipid bilayers had high membrane-integrity/sealing properties [37,38]. In more detail, Table 1 summarizes the full set of electrochemical parameters that were obtained by fitting the EIS data to the equivalent circuit model and confirmed that the fitted parameter values were in good agreement with expected ranges.

More specifically, the fitted G_m_ signal for the *E. coli* tBLM platform was around 1.36 ± 0.45 μS, which agrees well with recently studied tBLM platforms composed of biologically relevant two-chain phospholipids or three-chain triglycerides [39]. Of note, the fitted C_m_ signal was 0.83 ± 0.17 μF/cm^2^, which is within the range reported for tBLM platforms composed exclusively of two-chain phospholipids (~1.2–1.4 μF/cm^2^) or three-chain triglycerides (~0.6 μF/cm^2^). Since membrane conductance (G_m_) is the inverse of membrane resistance (R_m_), the obtained G_m_ range translates into R_m_ values of 820 ± 280 kΩ. Compared to tBLMs composed of two-chain phospholipids only, the lower C_m_ signal for the *E. coli* tBLM platform is indicative of a more densely packed membrane. This finding is consistent with the inclusion of four-chain CL lipids in the membrane composition as described above. 

In addition, the Q_s_ parameter represents the imperfect capacitance in the reservoir region, whereas the ∝_s_ parameter is defined as the CPE dimensional constant and describes the contribution of restricted ion diffusion in the reservoir region. The fitted values of both parameters agree well with those obtained for tBLM platforms composed of biologically relevant phospholipids [39], while the electrolyte resistance (R_e_) is also consistent with the ionic strength of the buffer composition used in this study. We also fabricated *E. coli* tBLM platforms from more purified polar lipid extracts, which had a similar lipid composition to the total extract and had been prepared by further precipitating the total lipid extract with acetone, followed by extraction with diethyl ether. In this case, similar electrochemical parameters were obtained, confirming that *E. coli* tBLM platforms could be fabricated from total or polar lipid extracts.

Since we were interested in characterizing how the tested mitigants affect tBLM properties, we focused on tracking quantitative changes in the G_m_ and C_m_ signals upon compound addition because these parameters are directly related to the membrane properties. Conversely, other fitted parameters are mainly related to the ionic reservoir space or bulk solution properties and are less directly affected by membrane properties. Practically, changes in the G_m_ and C_m_ signals were measured relative to their baseline values prior to compound addition in order to assess the interaction kinetics and corresponding degree of membrane disruption. Bode plot representations of the EIS frequency vs. phase were also analyzed in order to detect qualitative changes in the frequency-at-minimum-phase and phase-at-minimum-phase signatures before and after compound addition for ~30 min. Such information provides insight into changes in membrane ionic transport properties and membrane densification/thinning, respectively [40].

Therefore, after tBLM fabrication, the appropriate test compound at a defined concentration was added to the measurement chamber by pipette injection. The compound was incubated with the tBLM platform for 30 min during this treatment stage and then a buffer washing step was performed to remove the test compound from the bulk solution. The time resolution of the data collection was around 3 min per data point. To confirm the sensing capabilities of the tBLM platform composed of the total *E. coli* lipid extract, we first tested the membrane-disruptive effects of sodium dodecyl sulfate (SDS) at concentrations above and below CMC (Figure S2). SDS was active only above its CMC, and the interaction kinetics and membrane-solubilizing behavior showed general agreement with past EIS measurements conducted on tBLM platforms composed of diphytanoyl lipids as well as with results obtained using other techniques like electron microscopy [38,41]. We proceeded to test the membrane-disruptive effects of the different medium-chain fatty acids and monoglycerides on the tBLM platform composed of the total *E. coli* lipid extract.

### 2.1. EIS Measurements with Medium-Chain Fatty Acids

We compared the membrane-disruptive effects of 10-carbon long CA and 12-carbon long LA at different bulk concentrations (approximately 4×, 2×, and 0.5×) relative to their respective CMC values. The reported CMC values of CA and LA in equivalent buffer conditions are 3500 μM and 900 μM, respectively, and were used as guides to define the specific test concentrations [42]. The EIS data for each test compound are presented below.

#### 2.1.1. Capric Acid

**Figure 2:**
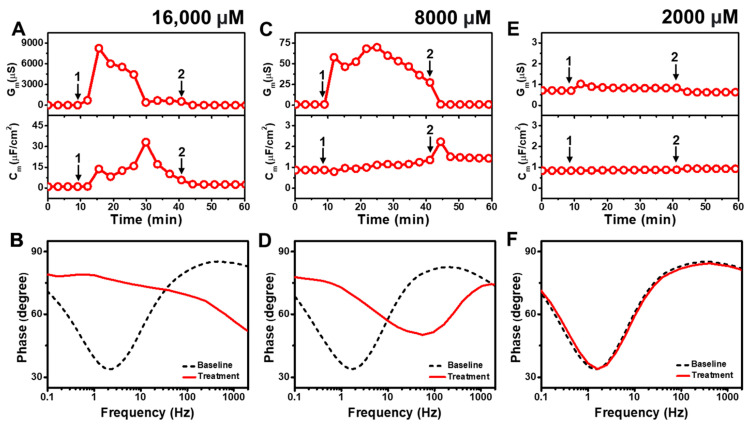
Time-resolved EIS measurements tracking effects of capric acid (CA) treatment on *E. coli* lipid-derived tBLM platform. (**A**) Conductance (G_m_) and capacitance (C_m_) signals are reported as a function of time for the addition of 16,000 μM CA (4× CMC) to tBLM platform at *t* = 10 min (arrow 1) and subsequent buffer rinsing step at *t* = 40 min (arrow 2). The baseline signals depict the tBLM platform prior to CA addition. (**B**) Bode plot snapshots for tBLM platform prior to CA addition and during CA treatment. (**C**–**F**) Corresponding data for 8000 μM CA (2× CMC) and 2000 μM CA (0.5× CMC) treatment cases. Graphs are representative from three independent runs.

Figure 2 presents the EIS results of CA addition to *E. coli* tBLMs at around 4×, 2×, and 0.5× CMC concentrations. Upon 16,000 μM CA addition to the tBLM (~4× CMC), the G_m_ and C_m_ signals initially spiked and reached maximum values around 8250 μS and 14 μF/cm^2^ (Figure 2A). Then, the G_m_ signal gradually decreased and eventually stabilized at around 675 μS, whereas the C_m_ signal increased transiently to a peak of ~33 μF/cm^2^ before gradually decreasing to ~17 μF/cm^2^. This dynamic interaction behavior indicates that CA addition causes a large increase in membrane conductance (and, thus, a decrease in membrane resistance) that is similar to the SDS effect (cf. Figure S2). However, in contrast to SDS, the CA interaction cannot lead to permanent membrane solubilization so the tBLM remodels itself over time to partially reorganize its sealing properties, albeit in a still highly damaged state. The final G_m_ and C_m_ values were reduced to around 1.6 μS and 2.5 μF/cm^2^ after buffer rinsing, pointing to a degree of membrane thinning that persisted even after CA was removed from the bulk solution. According to Bode plot analysis, 16,000 μM CA addition caused a shift in the EIS phase profile that indicated a surfactant-type interaction, which is consistent with the appreciable G_m_ shift that signified extensive membrane disruption during the interaction stage [43] (Figure 2B).

Upon 8000 μM CA addition (~2× CMC), the G_m_ signal increased to ~70 μS before gradually decreasing to roughly 27 μS (Figure 2C). A corresponding increase in the C_m_ signal of around ~1.2 μF/cm^2^ was also recorded. Subsequent buffer rinsing caused the G_m_ signal to decrease back to ~0.9 μS, while the C_m_ signal transiently spiked before remaining steady at around ~1.4 μF/cm^2^. The corresponding Bode plot showed that 8000 μM CA addition caused the phase minimum to shift to an appreciably higher frequency and phase, which corresponds to membrane damage and thinning (Figure 2D). By contrast, when 2000 μM CA was added (~0.5× CMC), there was only a slight rise in the G_m_ signal by around ~0.4 μS and negligible change in the C_m_ signal (Figure 2E). After buffer washing, the G_m_ signal returned to almost its baseline value and the C_m_ shift was insignificant. The Bode plot also showed negligible change in the phase minimum due to 2000 μM CA addition, supporting the idea that CA was inactive below its CMC (Figure 2F).

#### 2.1.2. Lauric Acid

**Figure 3:**
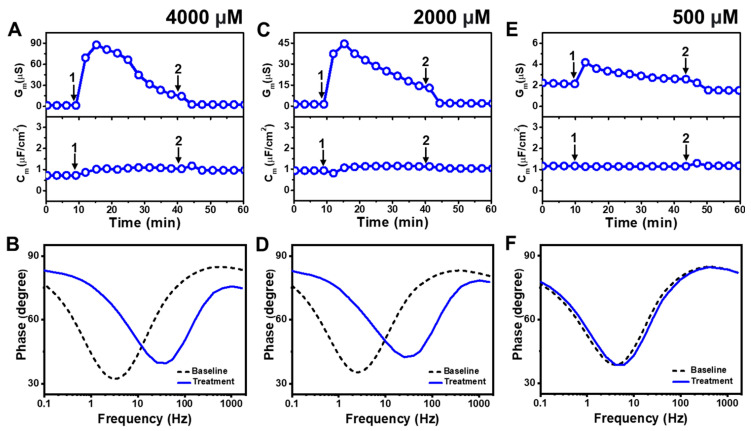
Time-resolved EIS measurements tracking effects of lauric acid (LA) treatment on *E. coli* lipid-derived tBLM platform. (**A**) Conductance (G_m_) and capacitance (C_m_) signals are reported as a function of time for the addition of 4000 μM LA (4× CMC) to tBLM platform at *t* = 10 min (arrow 1) and subsequent buffer rinsing step at *t* = 40 min (arrow 2). The baseline signals depict the tBLM platform prior to LA addition. (**B**) Bode plot snapshots for tBLM platform prior to LA addition and during LA treatment. (**C**–**F**) Corresponding data for 2000 μM LA (2× CMC) and 500 μM LA (0.5× CMC) treatment cases. Graphs are representative from three independent runs.

Figure 3 presents the EIS results of LA addition to *E. coli* tBLMs at around 4×, 2×, and 0.5× CMC concentrations. Upon 4000 μM LA addition to the tBLM (~4× CMC), the G_m_ and C_m_ signals transiently increased to ~88 μS and ~1 μF/cm^2^, respectively, while the G_m_ signal then gradually decreased to ~23 μS (Figure 3A). After buffer rinsing, the final G_m_ and C_m_ signals were reduced to around ~2.5 μS and ~1 μF/cm^2^, respectively. The corresponding Bode plots showed that the phase minimum transitioned to a modestly higher frequency and phase due to 4000 μM LA addition, which signified membrane damage (Figure 3B).

The interaction kinetics of the G_m_ signal response were similar in response to 2000 μM LA addition (~2× CMC), in which case there was an initial, transient increase to ~45 μS before gradually decreasing to ~18 μS (Figure 3C). After buffer rinsing, the G_m_ signal dropped to ~2 μS while the C_m_ signal shift was minimal throughout the interaction process. A similar change in the position of the phase minimum in the Bode plot was observed upon 2000 μM LA addition as in the 4000 μM LA addition case described above (Figure 3D). This finding indicates that 2000 μM LA addition also causes membrane damage, while the absence of a C_m_ signal shift supports that membrane disruption mainly stems from ionic permeability changes rather than from the loss of membrane integrity. On the other hand, the addition of 500 μM LA (~0.5× CMC) caused a much slighter and reversible change in the G_m_ signal to around ~4 μS and there was no change in the C_m_ signal (Figure 3E). The corresponding Bode plot revealed negligible membrane disruption upon 500 μM LA addition, with no change in the phase minimum that confirmed LA was inactive below its CMC (Figure 3F).

### 2.2. EIS Measurements with Medium-Chain Monoglycerides

Similar EIS experiments were performed to test the membrane-disruptive effects of 10-carbon long MC and 12-carbon long GML at different bulk concentrations (approximately 4×, 2×, and 0.5×) relative to their respective CMC values. The reported CMC values of MC and GML in equivalent buffer conditions are 600 μM and 60 μM, respectively, and were used as guides to define the specific test concentrations [42]. The EIS data for each test compound are presented below.

#### 2.2.1. Monocaprin

**Figure 4:**
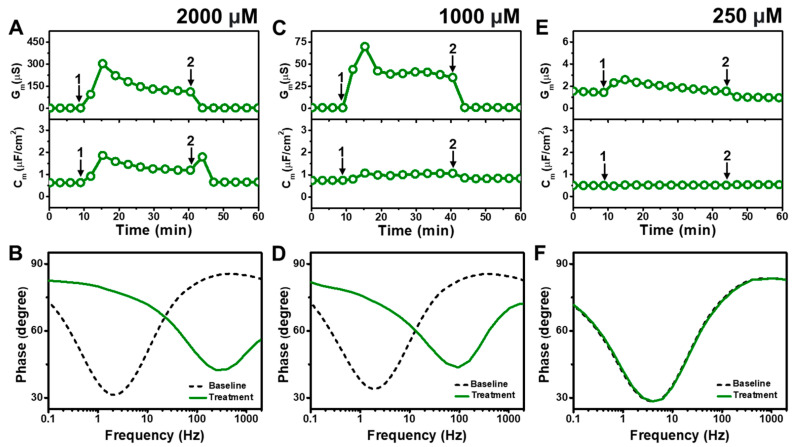
Time-resolved EIS measurements tracking effects of monocaprin (MC) treatment on *E. coli* lipid-derived tBLM platform. (**A**) Conductance (G_m_) and capacitance (C_m_) signals are reported as a function of time for the addition of 2000 μM MC (4× CMC) to tBLM platform at *t* = 10 min (arrow 1) and subsequent buffer rinsing step at *t* = 40 min (arrow 2). The baseline signals depict the tBLM platform prior to MC addition. (**B**) Bode plot snapshots for tBLM platform prior to MC addition and during MC treatment. (**C**–**F**) Corresponding data for 1000 μM MC (2× CMC) and 250 μM MC (0.5× CMC) treatment cases. Graphs are representative from three independent runs.

Figure 4 presents the EIS results of MC addition to *E. coli* tBLMs at around 4×, 2×, and 0.5× CMC concentrations. Upon 2000 μM MC addition to the tBLM (~4× CMC), the G_m_ and C_m_ signals rose to ~302 μS and ~1.9 μF/cm^2^, respectively, before stabilizing at around 123 μS and 1.2 μF/cm^2^, respectively (Figure 4A). The G_m_ and C_m_ signals then decreased to around 1.8 μS and 0.7 μF/cm^2^, respectively, after buffer rinsing. According to Bode plot analysis, extensive membrane damage occurred due to 2000 μM MC addition, as indicated by shifting of the phase minimum to a much higher frequency and phase (Figure 4B). 

With similar interaction kinetics, the addition of 1000 μM MC (~2× CMC) caused the G_m_ signal to increase to around 75 μS before stabilizing at around 41 μS (Figure 4C). However, there was only a slight and nearly negligible increase in the C_m_ signal. After buffer washing, the G_m_ and C_m_ signals returned to near-baseline values of around 1.0 μS and 0.8 μF/cm^2^, respectively. The Bode plots showed that 1000 μM MC addition caused the phase minimum to shift to a higher frequency and phase, albeit with a lower frequency shift than in the case of 2000 μM MC addition (Figure 4D). This finding supports that 1000 μM MC treatment still caused membrane damage, but to a lesser extent than 2000 μM MC treatment. By contrast, the addition of 250 μM MC (~0.5× CMC) caused only a slight increase in the G_m_ signal to around 1.8 μS, which returned to the baseline value following buffer washing (Figure 4E). No change in the C_m_ signal was observed as well. In addition, the Bode plots showed no change in the phase minimum before and after 250 μM MC addition, indicating negligible membrane-disruptive effects of MC below its CMC (Figure 4F). 

#### 2.2.2. Glycerol Monolaurate

**Figure 5:**
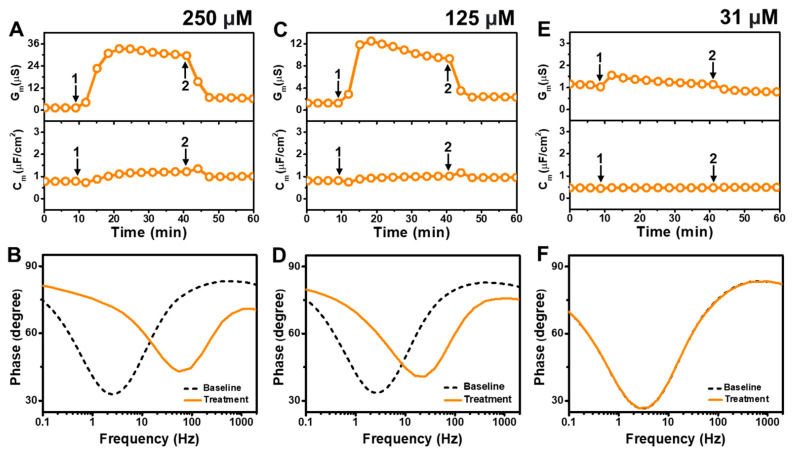
Time-resolved EIS measurements tracking effects of glycerol monolaurate (GML) treatment on *E. coli* lipid-derived tBLM platform. (**A**) Conductance (G_m_) and capacitance (C_m_) signals are reported as a function of time for the addition of 250 μM GML (4× CMC) to tBLM platform at *t* = 10 min (arrow 1) and subsequent buffer rinsing step at *t* = 40 min (arrow 2). The baseline signals depict the tBLM platform prior to GML addition. (**B**) Bode plot snapshots for tBLM platform prior to GML addition and during GML treatment. (**C**–**F**) Corresponding data for 125 μM GML (2× CMC) and 31 μM GML (0.5× CMC) treatment cases. Graphs are representative from three independent runs.

Figure 5 presents the EIS results of GML addition to *E. coli* tBLMs at around 4×, 2×, and 0.5× CMC concentrations. The addition of 250 μM GML (~4× CMC) caused the G_m_ signal to increase appreciably to ~31 μS while there was only a slight increase in the C_m_ signal to 1.2 μF/cm^2^ (Figure 5A). After buffer washing, the G_m_ and C_m_ signals decreased to 6.2 μS and ~1.0 μF/cm^2^, respectively. The Bode plots indicated that 250 μM GML caused the phase minimum to shift to a higher frequency and phase, which provided additional evidence of membrane damage (Figure 5B). 

In the case of 125 μM GML addition (~2× CMC), the G_m_ signal increased to around 10 μS, while the C_m_ signal only increased marginally to ~1 μF/cm^2^ (Figure 5C). After buffer washing, the G_m_ and C_m_ signals were reduced to around 2.3 μS and ~0.8 μF/cm^2^, respectively. The Bode plots before and after 125 μM GML addition showed a shift of the phase minimum to higher frequency and phase that indicated membrane damage, while the shift magnitudes were smaller than in the 250 μM GML case and, thus, demonstrate that the extent of membrane disruption was smaller in the 125 μM GML case (Figure 5D). We also tested the effects of 31 μM GML addition (~0.5× CMC) and observed only a modest increase in the G_m_ signal to around 1.2 μS, which returned to the baseline following buffer washing (Figure 5E). There was no change in the C_m_ signal as well. Furthermore, the Bode plot analysis showed that 31 μM GML addition caused no change in the position of the phase minimum, indicating that GML does not cause membrane disruption below its CMC (Figure 5F).

### 2.3. Comparison of Membrane-Disruptive Effects

**Figure 6:**
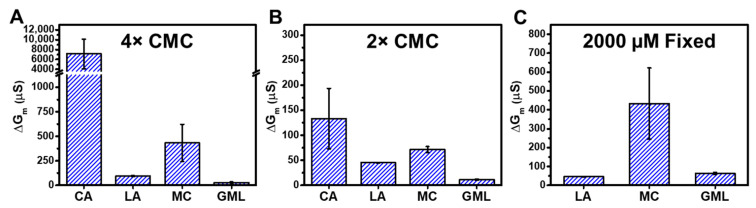
Effects of fatty acid and monoglyceride mitigant treatment on the electrochemical properties of reconstituted tethered *E. coli* membranes. The summarized maximum shifts in electrical conductance (ΔG_m_) are presented due to mitigant treatment at (**A**) 4× CMC, (**B**) 2× CMC, and (**C**) 2000 µM fixed concentration. The measurement data are reported as mean ± standard deviation from three independent replicates.

Figure 6 summarizes the trend in ΔG_m_ shifts that occurred when tBLM platforms composed of *E. coli* total lipid extracts were treated with different fatty acid and monoglyceride mitigants at 4× and 2× concentration levels relative to their respective CMCs. At 4× CMC, CA caused the largest ΔG_m_ shifts around 7100 μS and MC caused the second largest ΔG_m_ shifts around 420 μS. By contrast, LA and GML caused ΔG_m_ shifts of around 90 μS and 30 μS, respectively (Figure 6A). Thus, the membrane-permeabilizing effects of the four mitigants occurred in the following sequence: CA > MC > LA > GML. The same trend was observed when comparing EIS data obtained at 2× CMC (Figure 6B). In that case, CA demonstrated strong membrane-disruptive effects, with ΔG_m_ shifts around 130 μS. MC also exhibited extensive membrane disruption and caused ΔG_m_ shifts around 65 μS. On the other hand, LA and GML caused appreciably smaller ΔG_m_ shifts of around 45 μS and 10 μS, respectively (see also Table S1 for a detailed summary).

Since all four tested mitigants principally exhibited membrane-disruptive activity above their respective CMC values, this comparative approach supports that CA and MC with 10-carbon long saturated chains cause more extensive membrane disruption—as indicated by ionic permeability changes—than LA and GML with 12-carbon long saturated chains. In terms of comparing EIS data at equivalent molar concentrations, we may further note that 2000 µM MC caused ΔG_m_ shifts around 420 μS, whereas 2000 µM LA and 2000 µM GML caused smaller ΔG_m_ shifts around 45 μS and 60 μS, respectively (Figure 6C; see also Figure S3 for 2000 µM GML data). At this concentration, CA is inactive since its CMC is around 3500 µM, whereas it is difficult to work with 12-carbon long LA and GML at higher concentrations due to solubility considerations. 

In addition to the ΔG_m_ shifts that provide insight into ionic permeability changes, we may also briefly comment on the trend in ΔC_m_ shifts that reflect the degree of membrane integrity. In general, 10-carbon long CA and MC induced discernible ΔC_m_ shifts that pointed to a loss of *E. coli* membrane integrity due to membrane disruption and/or thinning [38]. By contrast, 12-carbon long LA and GML had a largely negligible effect on the ΔC_m_ shifts, indicating that membrane integrity was preserved even when there were modest permeability changes. 

We also proceeded to test the effects of the medium-chain fatty acids and monoglycerides on the tBLM platform derived from the polar *E. coli* lipid extract. Similar trends in ΔG_m_ and ΔC_m_ shifts were obtained compared to the data from the total *E. coli* tBLM platform, reinforcing that CA and MC with 10-carbon long saturated chains caused more extensive disruption of reconstituted *E. coli* membranes than LA and GML with 12-carbon long saturated chains (Figure S4).

As such, the overall difference in the membrane-disruptive effects of the tested mitigants with 10- vs. 12-carbon long, saturated hydrocarbon chains is consistent across the different EIS readouts and the variation in disruption extent may relate to the chain length-dependent packing parameters of the different mitigants. In general, the insertion of single-chain fatty acids and monoglycerides into phospholipid membranes can trigger spontaneous bilayer curvature [44]. With decreasing chain length, the hydrophobic part of the inserting, single-chain molecule is smaller and can, consequently, increase the degree of positive spontaneous curvature due to the membrane bending outward [44]. This bending affects inter-leaflet coupling and causes the bilayer core to become more disordered as compensation, which, in turn, leads to membrane thinning [44]. Conceptually, the induction of positive spontaneous curvature and membrane thinning would occur to a greater extent for CA compared to LA and for MC compared to GML, which matches with the experimental data trendwise and can be rationalized by the shorter chain lengths of CA and MC.

From a biological perspective, the EIS findings also agree well with the known antimicrobial spectrums of the different compounds; i.e., antimicrobial fatty acids and monoglycerides that cause greater membrane disruption are more likely to inhibit *E. coli,* and vice versa. Indeed, LA and GML are known to be among the most potent antimicrobial fatty acids and monoglycerides to inhibit Gram-positive bacteria, respectively, but are largely inactive against Gram-negative bacteria, especially *E. coli* [45,46,47,48,49,50]. In the EIS measurements, we observed that they cause smaller changes in membrane ionic permeability and do not affect the structural integrity of the *E. coli* membranes overall. In marked contrast, CA and MC have been reported to exhibit antibacterial activity against *E. coli* and cause bacterial cell damage [46,47,49,51,52], which are consistent with the appreciably larger changes in membrane ionic permeability as well as with the loss of membrane integrity and membrane thinning effects detected in the EIS measurements. Notably, it has been reported that CA causes greater in vitro membrane permeabilization of *E. coli* cell membranes than LA [49], which directly matches our results obtained with reconstituted *E. coli* membranes in the tBLM platform. Together, our findings support that studying the membrane-disruptive effects of fatty acid and monoglyceride mitigants with this bacterial lipid-derived EIS measurement approach can provide predictive insight into the antibacterial activity and potency of different test compounds to inhibit bacteria.

## 3. Materials and Methods

### 3.1. Materials

*E. coli* total lipid (no. 100500) and *E. coli* polar lipid (no. 100600) extracts, both dissolved in chloroform, were acquired from Avanti Polar Lipids, Inc. (Alabaster, AL, USA). According to the manufacturer’s specifications, the composition of the total lipid extract is 57.5% phosphatidylethanolamine (PE), 15.1% phosphatidylglycerol (PG), 9.8% cardiolipin (CL), and 17.6% other lipid components. The total lipid extract had been obtained as a chloroform:methanol extract from *E. coli* bacteria (ATCC 11303 strain), which was then participated against deionized water, and the extract corresponds to the concentrated chloroform phase [9]. The polar lipid extract is obtained by further precipitating the total lipid extract with acetone followed by extraction with diethyl ether, and its composition includes 67.0% PE, 23.2% PG, and 9.8% CL. Sodium dodecyl sulphate (SDS), capric acid (CA), monocaprin (MC), and lauric acid (LA) were obtained from Sigma Aldrich (St. Louis, MO, USA) and glycerol monolaurate (GML) was procured from Abcam (Cambridge, UK). Ultrapure water (>18.2 MΩ.cm resistivity) was obtained using a Milli-Q water purification system (MilliporeSigma, Burlington, MA, USA) and was used to prepare all buffer samples.

### 3.2. Mitigant Preparation

Ethanolic stock solutions of fatty acids and monoglycerides were prepared at 500 mM concentration by weighing out the desired mass of lyophilized sample, which was then dissolved in ethanol. Aliquots of the stock solutions were then diluted in phosphate-buffered buffer (pH 7.4, PBS) to the highest test concentration. Next, the experimental samples were heated at 70 °C for 30 min and, subsequently, allowed to cool to room temperature, followed by performing serial dilutions to prepare the test compounds at desired concentrations.

### 3.3. Electrochemical Impedance Spectroscopy (EIS) 

Electrochemical impedance spectroscopy (EIS) measurements were carried out using the SDx tethaPOD instrument (SDx Tethered Membranes, Sydney, Australia). A six-channel tethaPLATE cartridge was used for all measurements and prepared as follows: A glass slide patterned with gold electrode contacts was supplied with a benzyl-disulfide ethylene glycol monolayer coating that consisted of 10% tether (benzyldisulphide polyethylene glycol phytanyl) and 90% spacer (hydroxyl-terminated benzyldisulphide tetra-ethylene glycol) molecules. The pre-coated slide was rinsed with ethanol and left to dry for ~2 min before being attached to the cartridge. Tethered bilayer lipid membranes (tBLMs) of the desired membrane composition were fabricated, as previously described [38]. Briefly, 8 µL of a 3 mM ethanolic lipid solution was added to each channel of the monolayer-functionalized glass surface, which was then rinsed thrice with 100 μL PBS to form the tBLM and remove excess lipid. Afterwards, the cartridge was inserted into the tethaPOD instrument and the experimental operation was run with an alternating current (AC) impedance reader operating at frequencies ranging from 0.1 Hz to 2000 Hz with 25 mV amplitude (peak-to-peak AC excitation) and no applied potential (zero bias). The frequency sweep was performed from 2000 Hz to 0.1 Hz in descending order, with five steps per decade and a total data collection time of ~3 min per cycle. Data collection and analysis were carried out using the TethaQuick software package (SDx Tethered Membrane, version no. v2.0.58), which can fit the EIS data (frequency-dependent impedance magnitude and phase) to an equivalent circuit model by a procedure based on the Levenberg–Marquardt algorithm [43]. The chosen equivalent circuit model represents the tBLM as a resistor and capacitor that is in series with a constant phase element (CPE) that describes the imperfect capacitance of the gold electrode interface [53] (ionic reservoir region) and with a resistor that describes the impedance of the bulk electrolyte solution, as previously described [39]. The use of a CPE in the equivalent circuit model has been justified in the context of a heterogenous distribution of conductive elements (e.g., defects) within the membrane [43,54,55].

To prepare lipid samples for tBLM fabrication, lipid extracts from *E. coli* were supplied in chloroform and the appropriate volume of the desired composition was transferred to a glass vial, followed by gentle drying with nitrogen gas to form a thin, dry lipid film. Ethanol was then used to solubilize the dry lipid film to prepare a 3 mM lipid solution in ethanol.

## 4. Conclusions

In this study, we have investigated the membrane-disruptive effects of various medium-chain fatty acid and monoglyceride mitigants using *E. coli* tBLM platforms. The label-free EIS measurement approach enabled us to determine that all tested mitigants are mainly active above their respective CMC values while key differences in the extent of membrane disruption were identified. CA and MC with 10-carbon long hydrocarbon chains caused appreciably larger changes in ionic permeability than LA and GML with 12-carbon long hydrocarbon chains, and also caused more extensive structural damage to the tethered lipid bilayers, as indicated by the time-resolved tracking of the tBLM platform’s electrical conductance and capacitance properties. Since changes in ionic permeability and membrane integrity directly contribute to the antibacterial activity of mitigants in this class, the EIS readouts provided mechanistic insight into the potential utility of these mitigants for disrupting *E. coli* membranes. This capability is an important advantage of the EIS approach compared to other measurement options like the quartz crystal microbalance-dissipation (QCM-D) technique, which is sensitive to changes in biomacromolecular mass and hydrodynamically coupled solvent mass due to three-dimensional membrane remodeling processes when these mitigants interact with supported lipid bilayers, for example, but QCM-D does not directly probe permeability changes or membrane integrity.

Notably, both CA and MC are known to inhibit *E. coli,* whereas GML and LA are inactive against *E. coli,* and the EIS results obtained in this study agree well with those previously reported antibacterial activities, because CA and MC caused greater disruption of *E. coli* membranes, whereas LA and GML were appreciably less disruptive. These findings establish that the EIS technique is sensitive not only for the detection of the concentration-dependent onset of membrane disruption by a particular compound but also for the evaluation of the relative magnitudes of membrane disruption comparatively across a panel of compounds. The latter insights are particularly valuable because they support that the degree of membrane permeability change caused by an interacting amphiphilic molecule is an important factor rather than merely whether permeabilization occurs. While additional factors like the complex architectural properties of bacterial cell walls (e.g., peptidoglycan layer) might also influence the degree of antibacterial activity, our findings suggest that direct testing of antimicrobial mitigants with reconstituted bacterial lipid membranes is an advantageous measurement option for the real-time tracking of biologically relevant, membrane permeability changes. As lipid extracts from different bacterial species become available, it will be useful to further test the membrane-disruptive properties of antimicrobial drug and mitigant candidates against various types of bacteria, especially within a broader framework of correlating biophysical insights with microbiological evaluation and to design tailored mitigant formulations with enhanced activities for targeted applications (e.g., for food safety or cellular agriculture). Building on these capabilities, in the future, we may further explore how other electrochemical biosensing techniques based on voltametric methods like ramped/pulsed amperometry and cyclic voltammetry can be integrated with bacterial lipid-derived tBLM platforms to study fundamental mechanistic aspects of membrane disruption, including how electroporation-related membrane defects might modulate membrane-disruptive behaviors [37].

## Figures and Tables

**Table 1:** Electrochemical parameters of *E. coli* tBLM platforms formed using total and polar lipid extracts. The parameters were obtained by fitting the EIS data to an equivalent circuit model and are defined as follows: electrolyte resistance (R_e_), imperfect capacitance in reservoir region (Q_s_), CPE dimensional constant of reservoir region (∝_s_), membrane conductance (G_m_), and membrane capacitance (C_m_). The data are reported as mean ± standard deviation from *n* = 44 and *n* = 31 independent replicates for total and polar *E. coli* lipid extracts, respectively.

Composition	R_e_ (Ω)	Q_s_ (μF/cm^2^)	∝_s_	G_m_ (μS)	C_m_ (μF/cm^2^)
Total *E. coli*	732 ± 252	11.4 ± 1.0	0.85 ± 0.03	1.36 ± 0.45	0.83 ± 0.17
Polar *E. coli*	747 ± 231	11.8 ± 1.4	0.84 ± 0.02	1.41 ± 0.41	0.94 ± 0.21

## Data Availability

The raw data required to reproduce these findings are available from the corresponding authors upon reasonable request.

## Supplementary Materials

The following supporting information can be downloaded at: https://www.mdpi.com/article/10.3390/molecules29010237/s1, Figure S1: Representative Bode and Nyquist plot representations for *E. coli* tBLM platform formed using total lipid extract; Figure S2: Time-resolved EIS measurements tracking effects of sodium dodecyl sulfate (SDS) treatment on total *E. coli* lipid-derived tBLM platform; Figure S3: Time-resolved EIS measurements tracking effects of 2000 µM glycerol monolaurate (GML) treatment on total *E. coli* lipid-derived tBLM platform; Figure S4: Comparison of EIS measurement responses using total and polar *E. coli* lipid-derived tBLM platforms; Table S1: Summary of EIS measurement responses.
