# Supplementary material for: Membrane-Disruptive Effects of Fatty Acid and Monoglyceride Mitigants on E. coli Bacteria-Derived Tethered Lipid Bilayers
Source: Molecules. 2024 Jan 1;29(1):237. doi: 10.3390/molecules29010237 (PMC10780109; doi:10.3390/molecules29010237)
Supplement: Supplementary file 1 [file molecules-29-00237-s001.zip › molecules-2796113-supplementary.pdf]

Supplementary Materials

Membrane-Disruptive Effects of Fatty Acid and Monoglyceride Mitigants on *E. coli* Bacteria-Derived Tethered Lipid Bilayers

Sue Woon Tan [1], Bo Kyeong Yoon [2,*] and Joshua A. Jackman [1,*]

[1] School of Chemical Engineering and Translational Nanobioscience Research Center, Sungkyunkwan University, Suwon 16419, Republic of Korea

[2] School of Healthcare and Biomedical Engineering, Chonnam National University, Yeosu 59626, Republic of Korea

[*] Correspondence: bkyoon@jnu.ac.kr and jjackman@skku.edu

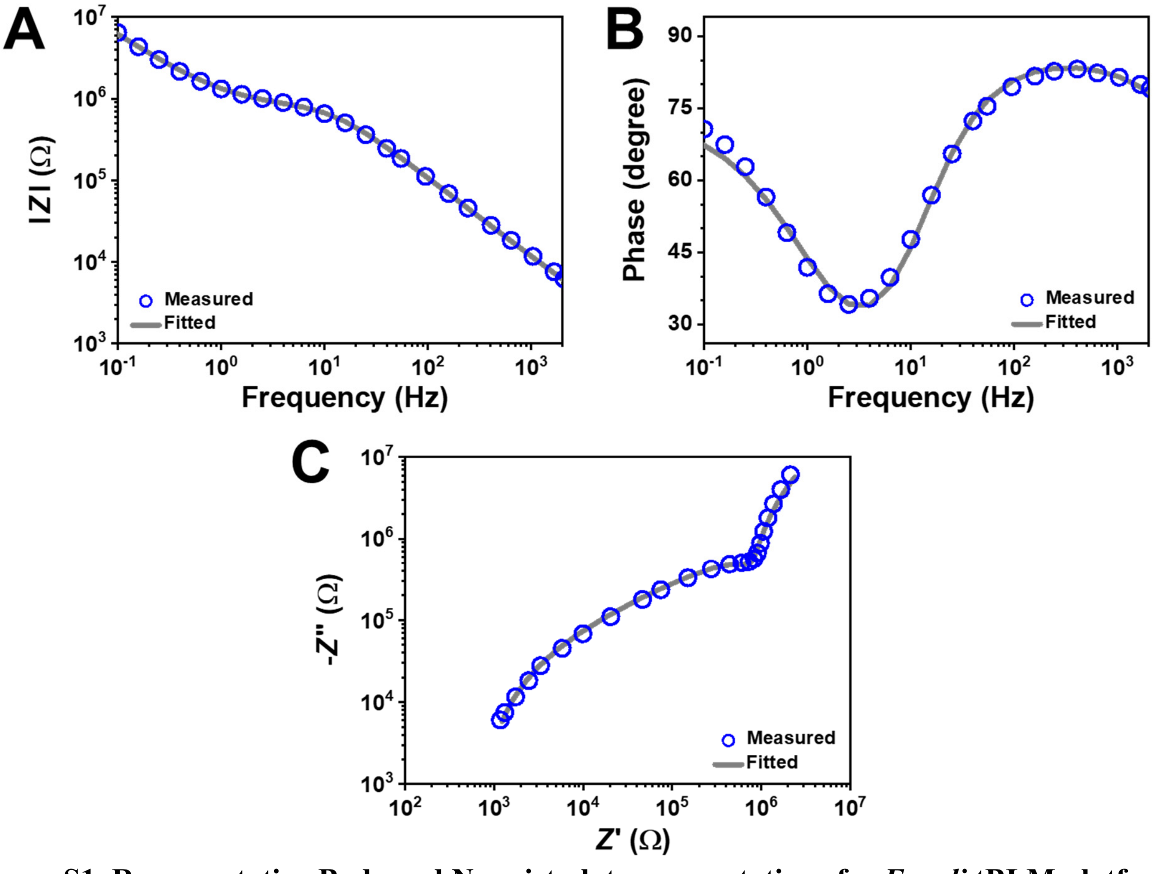

Figure S1. Representative Bode and Nyquist plot representations for *E. coli* tBLM platform formed using total lipid extract. Bode plot representations for **(A)** magnitude of total impedance (defined as |*Z*|) *vs.* frequency and **(B)** phase *vs.* frequency. Nyquist plot representation for **(C)** imaginary part of impedance (defined as *Z*''; plotted as -*Z*'') *vs.* real part of impedance (defined as *Z*'). Blue circles are experimental data points from EIS frequency sweep measurements and gray lines are drawn from calculated best-fit values that were obtained from fitting the data to an equivalent circuit model.

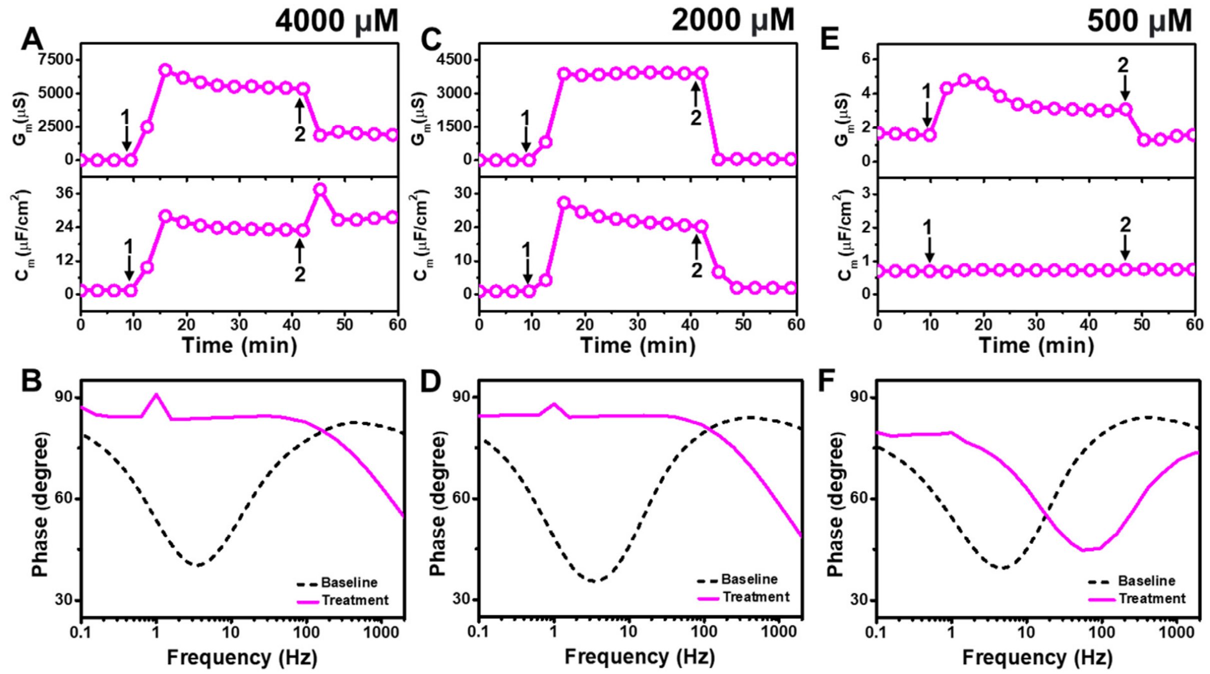

Figure S2. Time-resolved EIS measurements tracking effects of sodium dodecyl sulfate (SDS) treatment on total *E. coli* lipid-derived tBLM platform. (A) Conductance (G_m) and capacitance (C_m) signals are reported as a function of time for the addition of 4000 µM SDS (4× CMC) to tBLM platform at t=10 min (arrow 1) and subsequent buffer rinsing step at t=40 min (arrow 2). The baseline signals depict the tBLM platform prior to SDS addition. **(B)** Bode plot snapshots for tBLM platform prior to SDS addition and during SDS treatment. **(C-F)** Corresponding data for 2000 µM SDS (2× CMC) and 500 µM SDS (0.5× CMC) treatment cases. Graphs are representative from three independent runs.

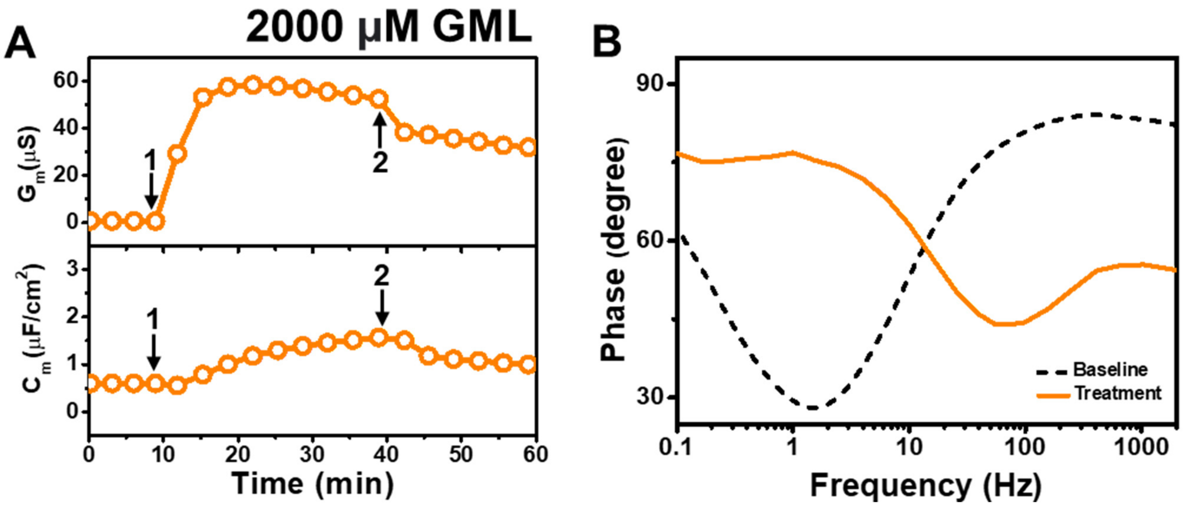

Figure S3. Time-resolved EIS measurements tracking effects of 2000 µM glycerol monolaurate (GML) treatment on total *E. coli* lipid-derived tBLM platform. (A) Conductance (G_m) and capacitance (C_m) signals are reported as a function of time for the addition of 2000 µM GML to tBLM platform at *t*=10 min (arrow 1) and subsequent buffer rinsing step at *t*=40 min (arrow 2). The baseline signals depict the tBLM platform prior to GML addition. **(B)** Bode plot snapshots for tBLM platform prior to GML addition and during GML treatment.

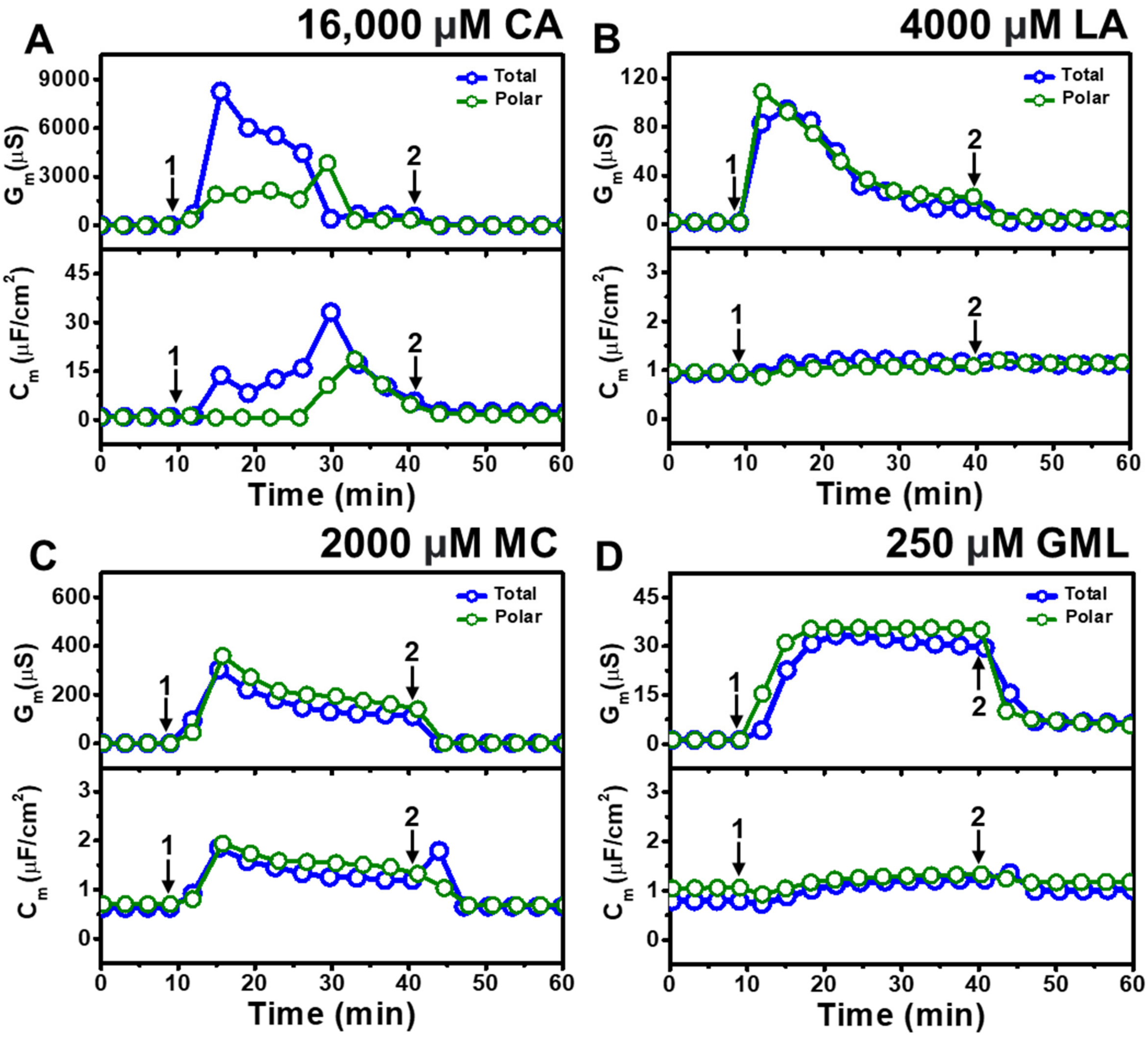

Figure S4. Comparison of EIS measurement responses using total and polar *E. coli* lipid-derived tBLM platforms. (A) Conductance (G_m) and capacitance (C_m) signals are reported as a function of time for the addition of 16,000 μM CA to tBLM platform at *t*=10 min (arrow 1) and subsequent buffer rinsing step at *t*=40 min (arrow 2). Blue and green circles signify the total and polar *E. coli* lipid extract cases, respectively. The baseline signals depict the tBLM platform prior to GML addition. **(B-D)** Corresponding data for 4000 μM LA, 2000 μM MC, and 250 μM GML treatment cases. Graphs are representative from three independent runs.

Table S1. Summary of EIS measurement responses. The maximum EIS responses upon compound addition at specified concentration to *E. coli* tBLM platform formed using total lipid extract are reported relative to baseline values. For comparison, the corresponding ΔG_m and ΔC_m shift values for 2000 μM GML (~32× CMC) were 62 ± 6 μS and 0.7 ± 0.1 μF/cm^2, respectively. In addition, at 0.5× CMC, the ΔG_m and ΔC_m shift values for all tested compounds were less than ~2 μS and ~0 μF/cm^2, respectively.

	**4× CMC**		**2× CMC**	
**Compound**	**ΔG_m (μS)**	**ΔC_m (μF/cm^2)**	**ΔG_m (μS)**	**ΔC_m (μF/cm^2)**
**SDS**	7408 ± 621	32 ± 4	4196 ± 295	27 ± 2
**CA**	7143 ± 3064	31 ± 11	133 ± 60	0.6 ± 0.2
**LA**	91 ± 3	0.3 ± 0.1	45 ± 1	0.2 ± 0.1
**MC**	422 ± 173	1.6 ± 0.5	67 ± 8	0.2 ± 0.1
**GML**	28 ± 4	0.1 ± 0.1	11 ± 1	0.1 ± 0.03
